# Critical carbon input to maintain current soil organic carbon stocks in global wheat systems

**DOI:** 10.1038/srep19327

**Published:** 2016-01-13

**Authors:** Guocheng Wang, Zhongkui Luo, Pengfei Han, Huansheng Chen, Jingjing Xu

**Affiliations:** 1 LAPC, Institute of Atmospheric Physics, Chinese Academy of Sciences, Beijing 100029, China; 2 CSIRO Agriculture, GPO Box 1666, Canberra, ACT 2601, Australia

## Abstract

Soil organic carbon (SOC) dynamics in croplands is a crucial component of global carbon (C) cycle. Depending on local environmental conditions and management practices, typical C input is generally required to reduce or reverse C loss in agricultural soils. No studies have quantified the critical C input for maintaining SOC at global scale with high resolution. Such information will provide a baseline map for assessing soil C dynamics under potential changes in management practices and climate, and thus enable development of management strategies to reduce C footprint from farm to regional scales. We used the soil C model RothC to simulate the critical C input rates needed to maintain existing soil C level at 0.1° × 0.1° resolution in global wheat systems. On average, the critical C input was estimated to be 2.0 Mg C ha^−1^ yr^−1^, with large spatial variability depending on local soil and climatic conditions. Higher C inputs are required in wheat system of central United States and western Europe, mainly due to the higher current soil C stocks present in these regions. The critical C input could be effectively estimated using a summary model driven by current SOC level, mean annual temperature, precipitation, and soil clay content.

Agricultural soils have been identified as having significant potential to sequester soil organic carbon (SOC) thereby mitigating climate change and maintaining soil productivity^1^,^2^. Significant efforts have been made to estimate the potential of agricultural soil to sequester C under diverse environmental and management conditions^3^,^4^,^5^,^6^. The actual amount sequestered depends on management strategies (e.g., residue retention and fertilizer application) and environmental conditions^7^,^8^,^9^. Regardless of management practices, increasing C input through residue retention and stimulating crop growth by nutrient intensification usually have to be achieved^10^,^11^,^12^,^13^. Across space and time, the required C input levels to maintain or increase soil C stock may vary widely depending on local soil and climatic conditions^9^,^11^,^14^. A detailed assessment of critical C input level targeting at certain soil C level will assist in designing effective management practices for C sequestration in agricultural soils and help different stakeholders assess the status of soil C under current farmers’ C input level and future potential management and climate changes.

In agricultural soils of different countries, soil C stock varies dramatically^15^. For example, in croplands of North American and European countries, the average SOC stock in the top 30 cm soil ranges from 56 to 67 Mg ha^−1^ and is comparable to or even exceeds the global average level of 54 Mg ha^−1^ 16,17. In China and India, however, the C stocks are only one third or two thirds of the global average^18^,^19^. This difference among countries is mainly due to cultivation history, land use intensity and agricultural management practices. Diverse soil (e.g., baseline SOC content) and climatic conditions are the other two most important factors influencing SOC stock, not only at the national level but also at the local scale. It is recognized that, under similar other environmental conditions, soils with higher SOC stock will require higher amount of C input to maintain the SOC level, and *vice versa*^14^,^20^,^21^. However, detailed estimation of required C input, at high resolution, is lacking across the world croplands, hindering development of specific management strategies to maximize the potential of agricultural soils to sequester C and assess the role of agro-ecosystems in climate change mitigation across scales.

Wheat is the third most produced cereals after maize and rice, and grown a larger area (~220 million hectares) than any other crops (http://faostat3.fao.org/home/E). Soil C dynamics in world wheat systems may substantially contribute to CO_2_ fluxes from global agro-ecosystems. As such, we focused on the SOC dynamics in world wheat systems in this study. The objectives of this study were to i) quantify the critical C input level to maintain current existing SOC level in world wheat systems at the resolution of 0.1° × 0.1° using a soil C model (RothC), ii) understand that how and why the critical C input level varies across space, and iii) develop a simple approach to estimate critical C input at finer scales.

## Results

### Performance of the RothC model

**Figure 1:**
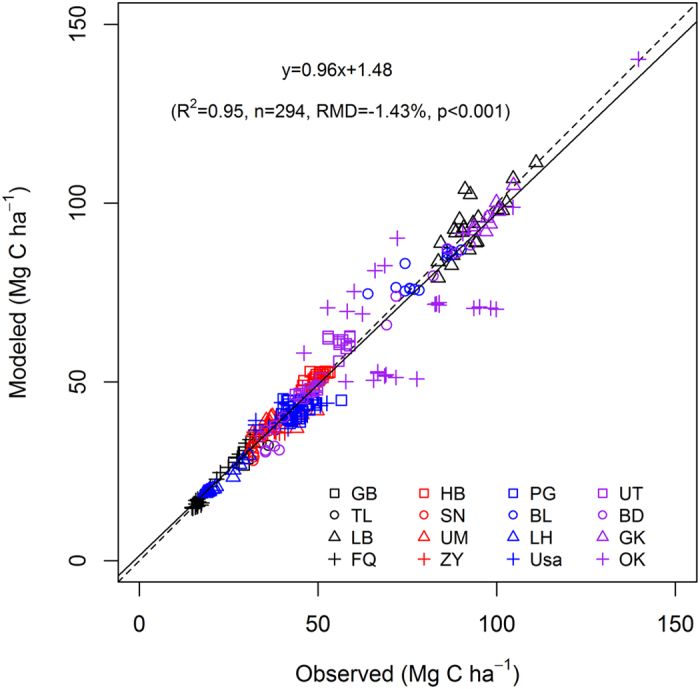
Modeled vs. observed SOC under 30 treatments at 16 sites. Dashed line is the 1:1 line. *n* is the sample size. RMD is the relative mean deviation. GB: Gibson; TL: Tarlee; LB: Lethbridge; FQ: Fengqiu; HB: Harbin; SN: Suining; UM: Urumuqi; ZY: Zhangye; PG: Prague; BL: Bad Lauchstädt; LH: Ludhiana; UT: Ultuna; BD: Broadbalk; GK: Grakov; OK: Oklahoma State Uni.

Among the 16 long-term experimental sites across the world’s wheat-growing regions (Fig. S1 and Table S1), the RothC model could generally simulate SOC change in the top 30 cm soil at most sites under various management (Fig. S2), with the exception at Urumuqi (Fig. S2g), Zhangye (Fig. S2h) and Prague (Fig. S2i). Pooling all data, the model explained 95% of the variation of observed SOC with a relative mean deviation (RMD) of −1.43% (Fig. 1). These results indicate that the RothC model initialized and parameterized using our approach (see Methods and Materials) is robust to predict SOC changes at different sites under different management in world wheat systems.

### Global pattern of critical C input to maintain SOC (C_maintain_)

**Figure 2:**
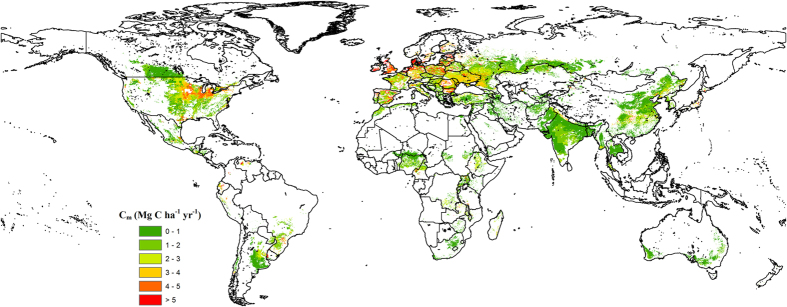
Critical carbon input rate to maintain current SOC level in world wheat systems. Map constructed in ESRI ArcMAP 10.1. Base image is obtained from the SEDAC database^44^.

Figure 2 shows the map of *C*_*maintain*_ in world wheat systems at the 0.1° × 0.1° resolution. Globally, average *C*_maintain_ was estimated to be 2.0 Mg C ha^−1^ yr^−1^, with the majority of *C*_maintain_ (~75% of the studied regions) is less than 3 Mg C ha^−1^ yr^−1^ except some areas in Europe (Fig. 2 and Table 1). Continentally, the average *C*_maintain_ in Europe was 3.1 Mg C ha^−1^ yr^−1^, and higher than that in Asia, Africa, South America, North America and Oceania (ranging from 1.1–2.2 Mg C ha^−1^ yr^−1^; Table 1). Nationally, the national average *C*_maintain_ was higher in the United States, France, Germany and Ukraine (e.g., ≥2.5 Mg C ha^−1^ yr^−1^) than that in the rest of the world’s 10 largest wheat-producing countries (Table 1).

### Variability of C_maintain_

**Figure 3:**
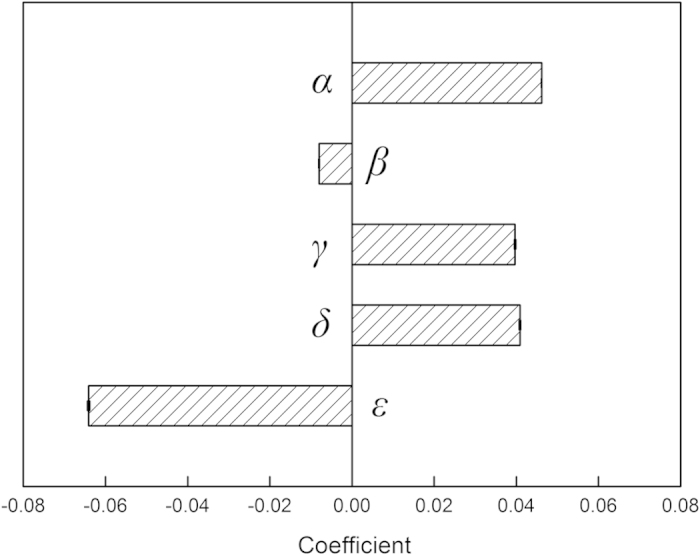
Coefficients for the regression model to predict critical C input to maintain current SOC (*C*_maintain_, Mg C ha^−1^ yr^−1^). The fitted model is: 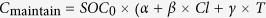

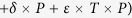
, where *SOC*_0_ is the current SOC stock (Mg C ha^−1^); *Cl, T* and *P* are the standardized (*z-score*) values of soil clay fraction (%), mean annual temperature (°C) and precipitation (m); *T* × *P* is the temperature-precipitation interaction. All the coefficients were significant at *P* < 0.0001, and the R^2^ of the whole model was 0.90. Error bars show 1 SE, which are very small for all the coefficients.

Across the world wheat-growing regions, large spatial variability existed in *C*_maintain_, and the variability could be explained by local-scale variables including the amount of current existing SOC stock (*SOC*_0_), soil clay content (*Clay*), mean annual temperature (*MAT*) and precipitation (*MAP*) (Fig. 3). Under otherwise similar environmental conditions, *C*_maintain_ increased with *SOC*_0_. Given *SOC*_0_ and *Clay*, *C*_maintain_ were higher in regions with higher temperature and rainfall. Given *SOC*_0_, *MAT* and *MAP*, however, *C*_maintain_ were higher in soils with lower clay fractions. The temperature-precipitation interaction also significantly influences the prediction of *C*_maintain_. Overall, *SOC*_0_ and its interaction with soil clay content, temperature and rainfall explained ~90% of the variation in the estimated *C*_maintain_ across world’s wheat systems (Fig. 3).

## Discussions

Our results provided a detailed map of the critical C input to maintain current C level in world wheat systems, and showed that the average global critical C input was 2.0 Mg C ha^−1^ yr^−1^ in order to maintain current SOC levels in world wheat systems, with a large spatial variability depending on soil and climatic conditions. The estimated critical C input positively correlated to current SOC stock, mean annual temperature and precipitation, but negatively to soil clay fraction. We suggested that the current SOC level could generally be maintained or even enhanced in most of the areas in Asia, North America, South America and Oceania under current C input management, because the current C input levels in these regions are generally higher than or equal to the estimated *C*_maintain_ (Table 1). In Europe and Africa, however, the wheat system may lose C under current management practices unless increasing C input. Special attention should be paid to Ukraine where current C input is only around two thirds of the estimated critical C input (Table 1). Clearly, additional input of 1.5 Mg C ha^−1^ yr^−1^ is required to cease soil C loss in Ukraine. If retaining all available C input (e.g. 100% residue retention) in the wheat system, all the top 10 wheat producing countries regions will be a C sink of atmospheric CO_2_, because the available C input levels in these regions are generally higher than the estimated *C*_maintain_ (Table 1).

The critical C input to maintain current soil C stocks in world wheat systems was significantly and positively related to the amount of current soil C stock (Fig. 3). This phenomenon is straight-forward as higher soil C means higher potential loss, and thus higher C input is required to keep the balance^20^,^22^. The effect of current soil C level was further strengthened by higher temperature and rainfall, suggesting that more C input is required in order to maintain C balance in wetter and warmer regions given a typical current C stock^23^. However, soil clay fraction has negative effect on required C input. This result confirms the finding that higher soil clay fraction could benefit C stabilization through mineralogical protection of soil C^24^,^25^.

It should be noted that the critical C input estimated in the present study can only be valid for the current existing SOC stock in wheat systems at the studied resolution. If the soil C varies over time and at finer spatial resolution, the critical C input needs to be re-quantified. The process-based models have the ability to predict SOC dynamics but normally require detailed information that is typically not readily obtainable at desired spatiotemporal resolution, thereby resulting in great challenges for decision-makers or potential users who have difficulty in using such models. Alternatively, a ‘space-for time’ substitution^26^ on the inputs and outputs of the process-based model simulations (meta-model) can help determine the relationship between the interests and their potential driving factors^27^. Such meta-model could then be used to predict critical C input under conditions when one or more of the covariates have changed. Here, we fitted the following meta-model to predict the RothC-modelled critical C input (*C*_maintain_, Mg C ha^−1^ yr^−1^) using current existing SOC stock (*SOC*_0_, Mg C ha^−1^), soil clay fraction (*Clay*, %), mean annual temperature (*MAT*, °C) and precipitation (*MAP*, mm):


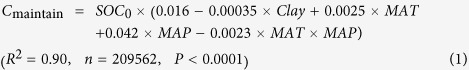


It should be noted that *C*_maintain_ was assumed not to be negative, and it should be set to zero if Eq. (1) yields a negative value. In general, this meta-model could explain 90% of the variations in *C*_maintain_ predicted by RothC, but required much less input information than RothC. This meta-model can be easily used as “space-for-time” substitution to infer critical C input under specific conditions. For example, irrigation was not taken into account in this study, causing potential underestimation of *C*_maintain_ in areas having irrigation. The effect of irrigation can be inferred by assuming that its effect is similar to rainfall.

Several uncertainties and limitations should be noticed in interpreting the simulation results in this study. First, the effect of tillage regimes on SOC were not simulated and it was assumed that the organic materials were incorporated into soils, due to the lacking of detailed information on tillage practice at global scale as well as the RothC model does not include a tillage module. However, our results would be still reasonable because existing studies have shown that conversion from conventional tillage to no-till has very limited ability to either accumulate SOC^28^ or mitigate climate change^29^ at global scales. Second, it has been reported that straw stoichiometry may affect SOC mineralization^30^. In the present study, we did not model the possible effect of nutrient availability on soil C dynamics because the RothC model does not have a functionality to simulate nitrogen dynamics. Ignoring the potential effect of straw stoichiometry on C decomposition may lead to biased estimation of *C*_maintain_ particularly in soils with low nutrient level^31^. Third, the RothC model does not include the processes or mechanisms involved with anaerobic respiration which might result in an overestimation of the *C*_maintain_ in some paddy soils across the eastern Asia, because the anaerobic conditions during rice growing season in paddy soils restricts SOC or straw decomposition. At last, it should be noted that although the RothC model could well simulate the observed SOC changes in most cases (Fig. S2), the model performed bad at Urumuqi, Zhangye and Prague. A possible reason is that large amounts of dust and other types of aerosols at these sites may affect temperature and precipitation and thus the model estimation of C dynamics which is mainly controlled by temperature and moisture^32^,^33^.

The spatially explicit estimates and high-resolution map of the critical C input generated in this study have important implications. First, the map can serve to assess the status of current C stock in world wheat systems under current management practices. This information is important to develop site-specific effective management strategies for soil C sequestration. Second, the map will help identify critical zones that have potential for carbon sequestration, and support national C accounting in wheat systems. In wheat systems of the USA, Russia and Ukraine, for example, the soil would be a continual source of atmospheric CO_2_ unless more residues are retained. Third, the derived meta-model can be readily used to infer C input requirement in order to maintain current soil C stock under future management and climate change. The model highlights the significance of the interaction between rainfall and temperature, implying the potential diverse consequences of global warming and rainfall change on soil C dynamics.

## Materials and Methods

The Rothamsted carbon model^34^ (RothC, version 26.3) was used to estimate C input rates to maintain the current soil C level in world wheat systems. RothC is a widely used soil organic matter (SOM) decomposition model to simulate C dynamics in agricultural soils under various environments and management^3^,^35^,^36^,^37^,^38^,^39^,^40^. The performance of the RothC model in simulating soil C dynamics was evaluated using observations obtained from 16 long-term experimental sites across the world’s wheat-growing regions (Fig. S1 and Table S1). Using the validated model, soil C dynamics between 2011 and 2050 under different C input scenarios were simulated at a resolution of 0.1° × 0.1°. For each of the 0.1° × 0.1° pixel, we identified the critical rates of C input to maintain the existing SOC stocks.

### Initialization and parameterization of the RothC model

In the RothC model, SOM is partitioned into five conceptual pools, i.e., decomposable plant material (DPM), resistant plant material (RPM), microbial biomass (BIO), humified organic matter (HUM), and inert organic matter (IOM). Such conceptual pools are not defined by direct measurable fractions, and are usually estimated by a spin-up run of the model to equilibrium or by physical fractionation. When applying RothC at large spatial scales (e.g. at the global scale in this study), both approaches to initialize the model are not possible because of limited data on physical fractions and the challenge of the required “run time” for the model spin-up. In this study, we adopted the approach of Falloon *et al.*^41^ and Weihermüller *et al.*^42^, who developed and validated a set of functions to initialize C pools in the RothC model:


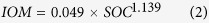



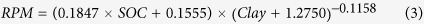



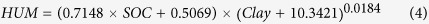



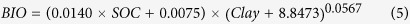


where SOC is the total soil organic C content in the 0–30 cm soil layer (Mg C ha^−1^) and clay is the soil clay fraction (%). The credibility of this approach was tested as described below.

For each of the datasets from the 16 long-term experiments, we tested the model performance using above functions (Eqns. 2–5) to initialize the sizes of C pools and the default decomposition rate constant of different C pools (i.e. 10.0 a^−1^ for DPM, 0.3 a^−1^ for RPM, 0.66 a^−1^ for BIO, 0.02 a^−1^ for HUM, and 0 for IOM). The default yearly rate constants were converted to monthly rate constants to allow the model to be run on a monthly time step. The annual C inputs for each experiment was either obtained from the corresponding literature (Table S1) or estimated based on crop yields using the approach of Huang *et al.* (2007). In determning the time of C input, we assumed that it occurs at the time of harvests. This setting is acceptable as RothC is known to be insensitive to the time of C input, particulary in long-term simulations^3^. The DPM/RPM ratio of the C input was set to 1.44, a typical value for most agricultural crops and grasses^34^.

In some experiments, SOC was only reported in the top 10 cm (*SOC*_*0–10cm*_) or 20 cm (*SOC*_*0–20cm*_) of soil. For these cases, the SOC stock in the top 30 cm soil layer (*SOC*_*0–30cm*_) was estimated based on the SOC vertical distribution assumption outlined in equations (6) to (8)^5^,^43^:


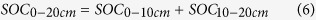



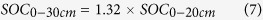



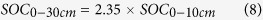


where 1.32 and 2.35 are the conversion coefficients.

### Global spatial datasets

The study area focused on the global wheat-growing regions. The map of global national boundaries was obtained from the NASA Socioeconomic Data and Applications Center (SEDAC)^44^. The geographic distribution of wheat-growing areas (0.1° × 0.1° spatial resolution, Fig. S1) and soil cover information were sourced from the Center for Sustainability and the Global Environment (SAGE)^45^. Soil parameters (0.1° × 0.1° resolution) including the clay content and SOC stock in the 0–30 cm soil layer were sourced from the Global Soil Dataset for use in Earth System Models (GSDE)^46^. The GSDE dataset is newly developed based on Soil Map of the World and various regional and national soil databases, it is the most comprehensive datasets of soil information at present^46^. Gridded climate projections (i.e. monthly mean temperature, precipitation and evaporation) at the resolution of 0.5° × 0.5° from 2011 to 2050 that produced by CESM1-CAM5-1-FV2 were extracted from the CMIP5 dataset^47^, and downscaled to the resolution of 0.1° × 0.1° using the method of Bertacchi *et al.*^48^.

We also derived the information on current C input (i.e. crop residues, roots and manure) rate from the FAOSTAT website (http://faostat3.fao.org/home/E), which was used to identify the feasibility of SOC sequestration under current C input levels (Table 1). The FAOSTAT online data contains crop-specific yield data across different spatial scales (e.g., national and continental). Following Huang *et al.*^49^, we assumed a constant Harvest index (0.4) and a constant root to top ratio (0.11) to calculate the quantity of crop residues and roots (e.g., available C input). According to published literature, the above-ground residue retention rates were assumed 30% in developing regions such as Asia and Africa^50^,^51^,^52^, and 60% in other regions^53^,^54^,^55^. The quantity of manure that were incorporated into soils across global croplands were estimated from the results of Potter *et al.*^56^. Based on these estimate of current C input, we also estimate the available C input (Table 1), assuming 100% residue retention.

### Model application to estimate the critical C input in world wheat systems

Using above data layers (wheat-growing regions, climate and soil), the RothC model was run in each of the 0.1° × 0.1° pixel under a C input scenario (from 0 to 10 Mg C ha^−1^ yr^−1^ in 0.1 Mg C ha^−1^increments) from 2011 to 2050. The SOC change (ΔSOC, Mg C ha^−1^) was calculated as the difference in the SOC between the final (SOC stock in 2050) and initial SOC stock (SOC stock in 2011). The lowest C input rate resulting in a positive ΔSOC was identified as the critical C input to maintain current existing SOC. This amount of C input was referred as *C*_maintain_. Then, *C*_maintain_ was mapped at the resolution of 0.1° × 0.1° and compared between major wheat-growing regions and countries.

### Meta-modeling to identify the drivers of C_maintain_

Meta-modelling is a sophisticated procedure to derive simple relationships from process-based modelling, and has been applied in soil C predictions^27^,^57^. The meta-model has the advantage of not needing detailed information to initialize process-based models and can be readily used by farmers and other policy-makers. Using the meta-modelling approach, we identified the impacts of climatic and soil variables on estimated *C*_maintain_. We hypothesized that the *C*_maintain_ was a function of current SOC stock, and environmental variables such as mean annual temperature (*MAT*) and precipitation (*MAP*) and soil clay fraction (*Clay*). We fitted the following model to assess the effect of current SOC stock (*SOC*_0_), and the regulations of *Clay*, *MAT* and *MAP* on this effect:


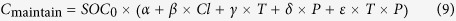


where *Cl, T* and *P* are the standardized (*z-score*) *Clay*, *MAT* and *MAP*, i.e., subtracting *Clay* (or *MAT* or *MAP*) at a given pixel by the mean in the whole study area and dividing by 2 standard deviations (*SD*); *α* is the predicted *C*_*maintain*_ (i.e., the slope) corresponding to a 1 Mg C ha^−1^ change in *SOC*_0_ when *Cl, T* and *P* are equal to zero, i.e., these variables are the same as the average of all pixels; *β*, *γ*, *δ*, and *ε* are the coefficients for *Cl*, *T*, *P* and *T* × *P* interaction, respectively. The *T* × *P* interaction could reflect the impact of particularly favorable or unfavorable combinations of temperature and rainfall^58^. The coefficients estimated based on *z-scores* have the advantage of indicating the relative importance of the predictors: more important with higher absolute values. Here, we did not fit an intercept for the model because when *SOC*_0_ was equal to 0, the *C*_maintain_ must also be 0 (i.e., no need of incorporating extra C). All analyses were performed with the statistical and graphical software R 3.0.2^59^.

## Additional Information

**How to cite this article**: Wang, G. *et al.* Critical carbon input to maintain current soil organic carbon stocks in global wheat systems. *Sci. Rep.* **6**, 19327; doi: 10.1038/srep19327 (2016).

## Supplementary Material

Supplementary Information

## Figures and Tables

**Table 1:** The C input, soil and climate information in global wheat-growing areas.

Region	C input (Mg ha^−1^)			C_0_^b^ (Mg ha^−1^)	MAT^c^ (°C)	MAP^d^ (mm)
	*C*_*maintain*_^a^	Current	Available			
Continent						
World	2.0 ± 1.6	2.2	4.7	54	13.9	0.8
Asia	1.2 ± 1.0	1.8	4.7	47	15.6	0.8
Europe	3.1 ± 1.8	2.6	5.3	67	9.2	0.6
Africa	1.5 ± 1.1	1.3	3.7	40	23.5	0.9
North America	2.2 ± 1.4	2.2	4.4	56	10.7	0.8
South America	1.7 ± 1.5	2.3	4.4	51	18.8	1.1
Oceania	1.1 ± 0.6	1.4	2.8	30	17.1	0.5
The top 10 wheat-producing countries						
China	1.5 ± 1.1	2.9	7.5	49	11.6	0.8
India	1.0 ± 0.9	2.1	4.9	37	26.1	1.1
USA	2.6 ± 1.4	2.2	4.5	58	12.1	0.8
Russia	1.9 ± 1.2	1.3	2.8	67	5.2	0.5
France	2.9 ± 1.3	4.7	9.9	60	11.8	0.8
Canada	1.3 ± 0.8	1.9	4.2	55	3.3	0.5
Germany	4.5 ± 2.5	5.3	10.8	82	9.6	0.7
Pakistan	0.8 ± 0.6	1.8	4.4	21	23.8	0.4
Australia	1.1 ± 0.6	1.4	2.7	30	17.2	0.5
Ukraine	3.6 ± 1.2	2.1	4.3	73	9.8	0.5

^a^Values show the mean ± SD of the model estimates in the corresponding region.

^b^The current existing soil organic carbon stock.

^c^The mean annual temperature.

^d^The mean annual precipitation.
