# Supplementary material for: Critical carbon input to maintain current soil organic carbon stocks in global wheat systems
Source: Sci Rep. 2016 Jan 13;6:19327. doi: 10.1038/srep19327 (PMC4725856; doi:10.1038/srep19327)
Supplement: Supplementary Information [file srep19327-s1.pdf]

Critical carbon input to maintain current soil organic carbon stocks in global wheat systems

Guocheng Wang[1,*], Zhongkui Luo[2], Pengfei Han[1], Huansheng Chen[1], Jingjing Xu[1]

Supplementary Figure 1 - Location of the long-term field experiments and distribution of wheat producing area. Map constructed in ESRI ArcMAP 10.1. Base image is obtained from the SEDAC database [1].

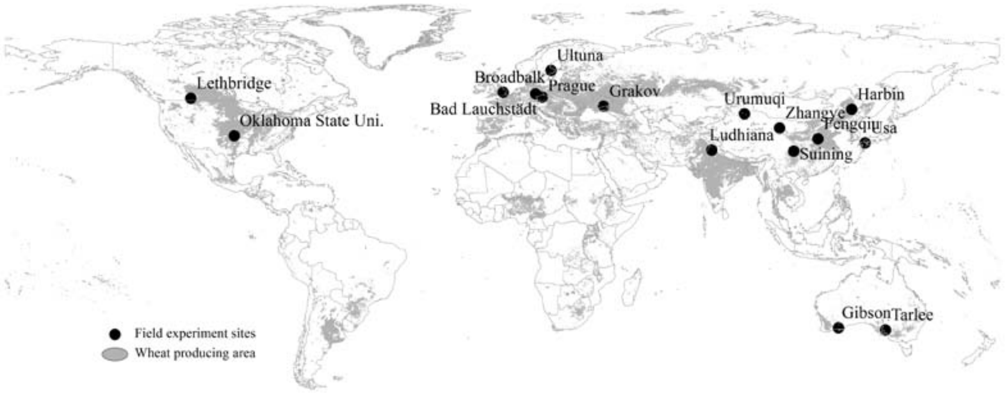

Supplementary Figure 2 - Simulated and observed SOC at different sites: Gibson (a), Tarlee (b), Lethbridge (c), Fengqiu (d), Harbin (e), Suining (f), Urumuqi (g), Zhangye (h), Prague (i), Bad Lauchstädt (j), Ludhiana (k), Usa (l), Ultuna (m), Broadbalk (n), Grakov (O) and Oklahoma State Uni. (p).

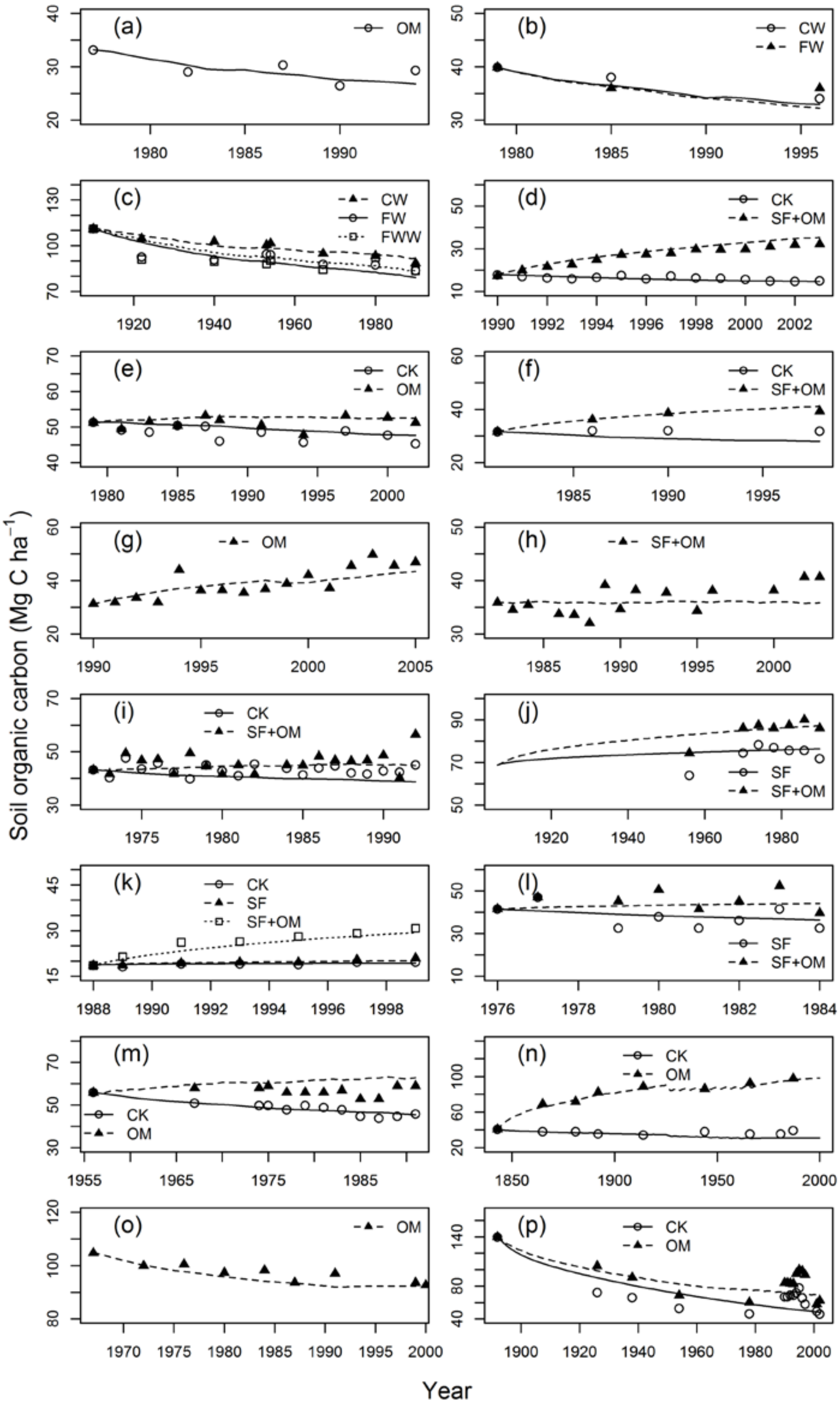

Supplementary Table 1 - Detailed information on the data of the long-term field experiments used for RothC validation.

Country	Site name	MAT[a] (℃)	MAP[b] (mm)	Clay (%)	SOC0[c] (Mg ha^{-1})	Time span	Treatment	Input C (Mg ha^{-1} yr^{-1})	Source
Australia	Gibson	16.6	476	7	33.12	1977-1994	OM[d]	0.77	Wang, et al. [2]
Australia	Tarlee	15.2	454	22	39.9	1979-1996	CW[e], FW[f]	0.72, 0.57	Wang, et al. [2]
Canada	Lethbridge	3.5	358	42	111	1910-1990	CW, FW, FWW[g]	1.8, 1.2, 0.9	Monreal and Janzen [3]
China	Fengqiu	13.9	605	9	17.7	1990-2003	CK[h], SF[i]+OM	0.2, 6.5	Yu, et al. [4]
China	Harbin	3.5	533	25	51.3	1979-2002	CK, OM	0.8, 1.5	Yu, et al. [4]
China	Suining	17.4	1014	24	31.6	1981-1998	CK, SF+OM	0.9, 4.1	Yu, et al. [4]
China	Urumuqi	7.7	310	21	31.2	1990-2005	OM	8.1	Yu, et al. [4]
China	Zhangye	7	127	12	35.9	1982-2003	SF+OM	1.9	Yu, et al. [4]
Czech	Prague	8.7	550	27	43.2	1972-1992	CK, SF+OM	0.9, 1.35	Whitmore, et al. [5]
Germany	Bad Lauchstädt	9.1	463	21	65.5	1906-1990	SF, SF+OM	2.6, 3.5	Coleman, et al. [6]
India	Ludhiana	24.9	800	13	18.7	1988-1999	CK, SF, SF+OM	1.4, 1.6, 4.4	Bhattacharyya, et al. [7]
Japan	Usa	15.5	1520	23.5	45.1	1976-1984	SF, SF+OM	0.5, 3.3	Shirato and Taniyama [8]
Sweden	Ultuna	5.5	537	37	55.8	1956-1991	CK, OM	0.5, 2.5	Karhu, et al. [9]
UK	Broadbalk	9.8	595	23	86.5	1876-1991	CK, OM	1.2, 4.9	Jenkinson, et al. [10]
Ukraine	Grakov	6.7	389	39	104.7	1967-1999	OM	3.1	Franko, et al. [11]
USA	Oklahoma State Uni.	15.9	873	20	142.6	1892-2002	CK, OM	0.2, 1.73	Davis, et al. [12]

[a] Mean annual temperature. [b] Mean annual precipitation. [c] Initial SOC density. [d] Organic matter. [e] Continuous wheat. [f] Fallow-wheat. [g] Fallow-wheat-wheat. [h] Control. [i] Synthetic fertilizer.

References

1. NASA Socioeconomic Data and Applications Center (SEDAC)., *Gridded Population of the World,* Version *3 (GPWv3): National Administrative Boundaries*. (2005) Available at: http://sedac.ciesin.columbia.edu/data/set/gpw-v3-national-%20admin-boundaries. (Accessed:12 May 2015).
2. Wang, G. C., Huang, Y., Wang, E. L., Yu, Y. Q. & Zhang, W. Modeling soil organic carbon change across Australian wheat growing areas, 1960–2010. *PLoS ONE* **8**, e63324, (2013).
3. Monreal, C. & Janzen, H. Soil organic-carbon dynamics after 80 years of cropping a Dark Brown Chernozem. *Can J Soil Sci* **73**, 133-136, (1993).
4. Yu, Y. Q., Huang, Y. & Zhang, W. Modelling soil organic carbon change in croplands of China, 1980-2009. *Global Planet Change* **82-83**, 115-128, (2012).
5. Whitmore, A. *et al.* Simulating trends in soil organic carbon in long-term experiments using the Verbeme/MOTOR model. *Geoderma* **81**, 137-151, (1997).
6. Coleman, K. *et al.* Simulating trends in soil organic carbon in long-term experiments using RothC-26.3. *Geoderma* **81**, 29-44, (1997).
7. Bhattacharyya, T. *et al.* Evaluating the Century C model using long-term fertilizer trials in the Indo-Gangetic Plains, India. *Agr Ecosyst Environ* **122**, 73-83, (2007).
8. Shirato, Y. & Taniyama, I. Testing the suitability of the Rothamsted carbon model for long-term experiments on Japanese non-volcanic upland soils. *Soil Sci Plant Nutr* **49**, 921-925, (2003).
9. Karhu, K. *et al.* Impacts of organic amendments on carbon stocks of an agricultural soil—comparison of model-simulations to measurements. *Geoderma* **189**, 606-616, (2012).
10. Jenkinson, D., Andrew, S., Lynch, J., Goss, M. & Tinker, P. The turnover of organic carbon and nitrogen in soil [and discussion]. *Philosophical Transactions of the Royal Society of London. Series B: Biological Sciences* **329**, 361-368, (1990).
11. Franko, U., Kuka, K., Romanenko, I. & Romanenkov, V. Validation of the CANDY model with Russian long-term experiments. *Regional Environmental Change* **7**, 79-91, (2007).
12. Davis, R. *et al.* Nitrogen balance in the Magruder plots following 109 years in continuous winter wheat. *J Plant Nutr* **26**, 1561-1580, (2003).
